# Unique Responses are Observed in Transient Receptor Potential Ankyrin 1 and Vanilloid 1 (TRPA1 and TRPV1) Co-Expressing Cells

**DOI:** 10.3390/cells3020616

**Published:** 2014-06-11

**Authors:** Laura R. Sadofsky, Koti T. Sreekrishna, Yakang Lin, Renee Schinaman, Kate Gorka, Yogita Mantri, John Christian Haught, Thomas G. Huggins, Robert J. Isfort, Charles C. Bascom, Alyn H. Morice

**Affiliations:** 1 Cardiovascular and Respiratory Studies, The University of Hull, HU6 7RX, UK; E-Mails: Sadofsky@hull.ac.uk (L.R.S.); A.H.Morice@hull.uk (A.H.M.); 2 The Procter & Gamble Company, Mason, OH 45040, USA; E-Mails: lin.y.2@Pg.com (Y.L.); schinaman.cr@pg.com (R.S.); gorka.k@pg.com (K.G.); mantri.y@pg.com (Y.M.); haught.c@pg.com (J.C.H.); huggins.tg@pg.com (T.G.H.); Isfort.rj@pg.com (R.J.I.); bascom.cc@pg.com (C.C.B.)

**Keywords:** TRPV1, TRPA1, TRPA1, TRPV1 co-expression

## Abstract

Transient receptor potential (TRP) ankyrin 1 (TRPA1) and vanilloid 1 (TRPV1) receptors are implicated in modulation of cough and nociception. *In vivo*, TRPA1 and TRPV1 are often co-expressed in neurons and TRPA1V1 hetero-tetramer formation is noted in cells co-transfected with the respective expression plasmids. In order to understand the impact of TRP receptor interaction on activity, we created stable cell lines expressing the TRPA1, TRPV1 and co-expressing the TRPA1 and TRPV1 (TRPA1V1) receptors. Among the 600 compounds screened against these receptors, we observed a number of compounds that activated the TRPA1, TRPV1 and TRPA1V1 receptors; compounds that activated TRPA1 and TRPA1V1; compounds that activated TRPV1 and TRPA1V1; compounds in which TRPA1V1 response was modulated by either TRPA1 or TRPV1; and compounds that activated only TRPV1 or TRPA1 or TRPA1V1; and one compound that activated TRPA1 and TRPV1, but not TRPA1V1. These results suggest that co-expression of TRPA1 and TRPV1 receptors imparts unique activation profiles different from that of cells expressing only TRPA1 or TRPV1.

## 1. Introduction

TRPA1 and TRPV1 have important roles in the sensation of pain, temperature, inflammation and cough in animals and man [1,2]. TRPV1 is activated by warm temperatures (above 43 °C), protons and noxious chemicals such as capsaicin and resiniferatoxin [3]. TRPA1 is activated by cold temperatures (below 17 °C), and a wide range of irritating and pain stimulating chemicals such as acrolein (found in smoke), formalin, mustard oil and allicin (found in onions and garlic) as well as cinnamaldehyde (extracted from cinnamon) [1,4,5,6].

Functional TRP channels are thought to be tetramers, possibly either homo-tetramers or even hetero-tetramers [7,8]. *In vivo*, TRPA1 is known to be expressed in the same sensory neurons as TRPV1 [5] and pharmacological interaction between the two receptors has been established [9,10,11,12]. Direct interaction resulting in hetero-tetramers between these two channels has been demonstrated using transient co-expression of the two receptors in CHO cells [13]. Furthermore, it has been demonstrated that TRPV1 regulates modulation of TRPA1 by Ca^2+^ [14]. In the present study, we have evaluated the response of cells stably co-expressing the TRPA1, TRPV1 and TRPA1V1 receptors to 600 compounds. In this report, we demonstrate that the response of TRPA1 and TRPV1 co-expressing cells to agonists shows expected and novel agonist specificity responses. 

## 2. Experimental Section

### 2.1. Materials

Allyl isothiocyanate (AITC), cinnamaldehyde, capsaicin, and calcium ionophores (A23187 and ionomycin) were obtained from Sigma Aldrich (St Louis, MO). All buffers, expression vectors, antibiotics, calcium dyes (Fluo-3 AM and Fluo-4 AM), and other reagents used were obtained from Life Technologies (Carlsbad, CA, USA). A compound library was procured from Evotec (San Francisco, CA, USA).

### 2.2. TRPV1, TRPA1, TRPA1V1 and pcDNA3 Control Cells

Cells stably expressing human TRPV1, human TRPA1, as well as cells that co-express both receptors (TRPA1V1) and control (pcDNA3) cells have been described previously [15,16,17]. Cells stably expressing TRPM8 and TRPV3 were obtained from Dr. Michael Xi Zhu, University of Texas Health Science Center, Houston, TX.

### 2.3. Measurement of Intracellular Calcium for Activation of TRA1V1, TRPA1 and TRPV1

TRPV1, TRPA1, TRPA1V1 and pcDNA3 cells were grown in 15 mL growth medium [high glucose DMEM (Dulbecco’s modification of Eagle’s medium) supplemented with 10% FBS (fetal bovine serum), 100 μg/mL penicillin/ streptomycin, 100 μg/mL G418] in a 75 cm^2^ flask for 3 days in a mammalian cell culture incubator at 33 °C and 5% CO_2_. TRP cells were detached with 8 mL of PBS (without calcium or magnesium); for pcDNA3 cells, trypsin was used for releasing the cells. The detached cells were spun at low speed (800–900 rpm for 3 min) to pellet the cells. The PBS medium was gently removed, and the cell pellet was re-suspended in 1 mL growth medium; 12.5 μg of Fluo-4 AM calcium dye dissolved in 5 μL Pluronic F-127 (20% solution in DMSO), was added and incubated for 30 min with gentle shaking at room temperature. The cells were washed once with 45 mL assay buffer (1× HBSS, 20 mM HEPES) by low speed centrifugation (800–900 rpm for 3 min) and resuspended in 11 mL of the assay buffer in a reagent reservoir. Aliquots of 100 μL (approximately 5 × 10^4^ cells) were dispensed in each well of the 96-well plate (BD Falcon micro-test assay plate #353948). The plates were set at room temperature for 30 min. The plates were read in a FLIPR^TETRA^ instrument (Molecular Devices, Sunnyvale, CA) at λ_ex_ 488 nm and λ_em_ 514 nm to record baseline fluorescence following which 20 μL of test material at 1 mM final concentration for pure compounds and 0.004% for extracts in the library. The agonists, capsaicin (350 nM) and AITC (30 μM); and controls, ionomycin (10 μM) and buffer alone, were added to each well using the dispenser provided in the FLIPR. The data point was recorded every 2 seconds for a total of 10 min. Data were analysed after baseline subtraction as describe previously [18,19]. 

## 3. Results and Discussion

### 3.1. Screening TRPA1V1, TRPA1 and TRPV1Receptor Containing Cells with 600 Compounds

#### 3.1.1. Cell Culture Conditions

**Figure 1:**
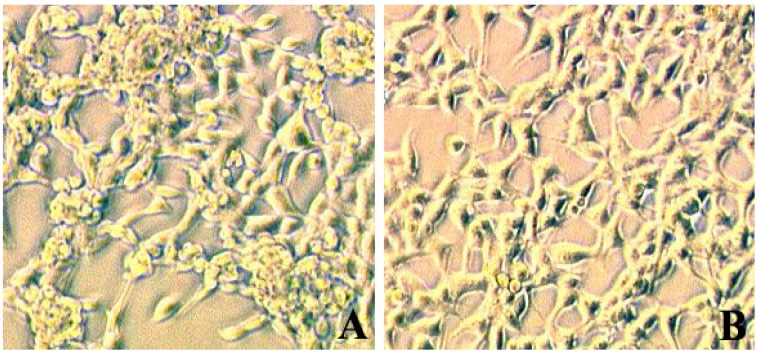
TRPV1 cells grown at 37 °C (**A**) and 33 °C (**B**).

**Figure 2:**
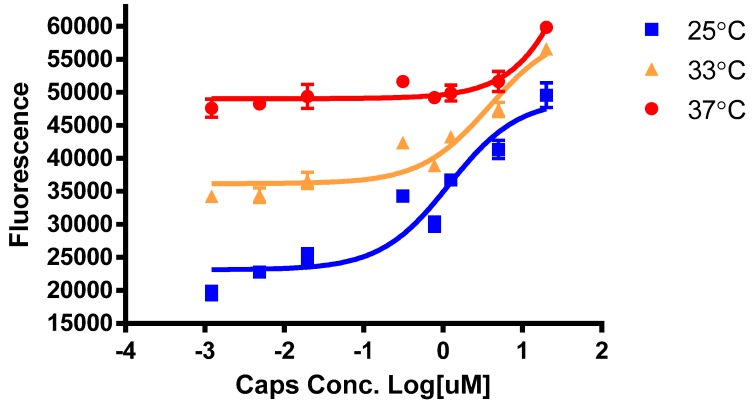
TRPV1 cells pre-incubated at 25 °C for 30 min, prior to addition of agonist, gives better response.

We observed that during cultivation of cells for several passages, TRPV1 cells cultivated at 37 °C formed clumps and appeared unhealthy presumably due to temperature induced high basal activity of TRPV1. However, cells grown at 33 °C did not form clumps and looked healthy (Figure 1). Pre-incubation of cells at 25 °C for 30 min prior to addition of capsaicin gave superior response as compared to cells pre-incubated at 33 °C or 37 °C (Figure 2). Cells pre-incubated at higher temperatures had proportionally higher basal activity. Thus, for screening compounds, we implemented these growth temperature and pre-incubation conditions for all three cell lines.

#### 3.1.2. Identification of TRPA1, TRPV1 and TRPA1V1 Agonists

We screened a library of 600 compounds (including extracts) in cells containing TRPA1, TRPV1 and TRPA1V1 channels in order to identify compounds that act as agonists at these channels. We excluded 22 compounds (including extracts) that activated Calcium-flux in pcDNA3 control cells. Compounds or extracts that gave at least a 20% increase in channel activity relative to benchmark agonist against TRPA1, TRPV1 or TRPA1V1 expressing cells were considered an agonist. From this screen we identified the following classes of TRPA1, TRPV1 and TRPA1V1 agonists [average value for activation from three independent assay plates are presented as % of agonist value (AITC, 30 μM for TRPA1 and capsaicin, 350 nM for TRPV1. For TRPA1V1, we used AITC, 30 μM or capsaicin, 350 nM depending on if the compound is a TRPA1 agonist or a TRPV1 agonist. For compounds that activated both TRPA1 and TRPV1, we used AITC for normalization). Variation among triplicates was within 10% of each other]. At the concentration of AITC used here, we did not encounter any non-specific effect as previously noted by others with AITC [20,21,22].

**Figure 3:**
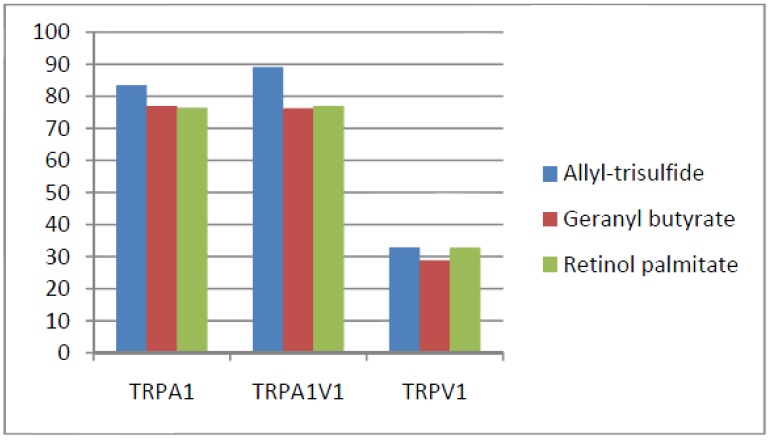
Examples of compounds that activated all three receptors (TRPA1 = TRPA1V1 > TRPV1).

**Figure 4:**
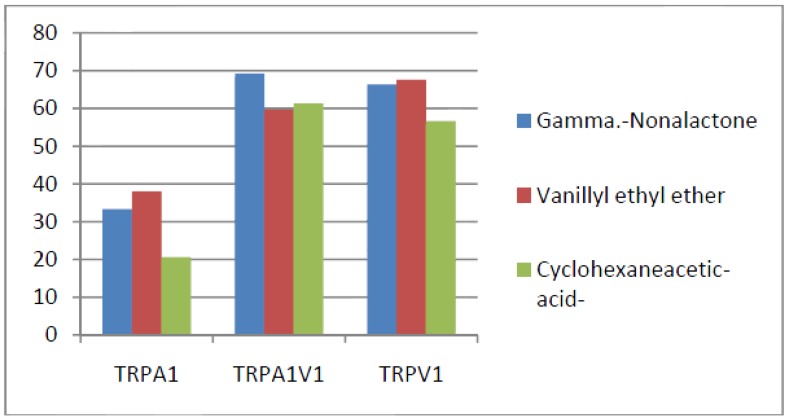
Examples of compounds that activated all three receptors (TRPA1 < TRPA1V1 = TRPV1).

**Figure 5:**
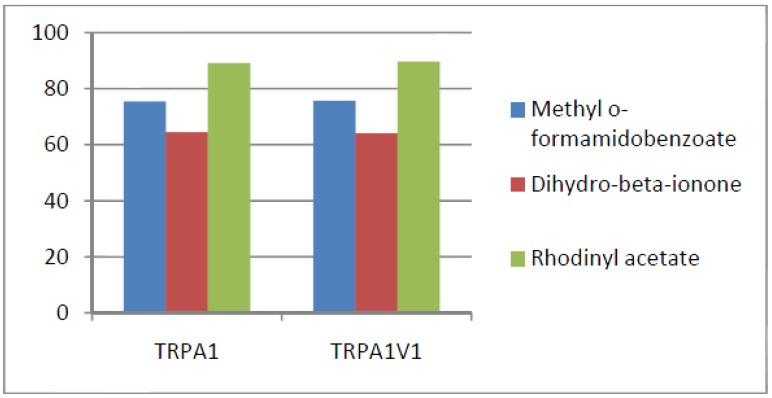
Examples of compounds that activate TRPA1 = TRPA1V1, but not TRPV1.

**Figure 6:**
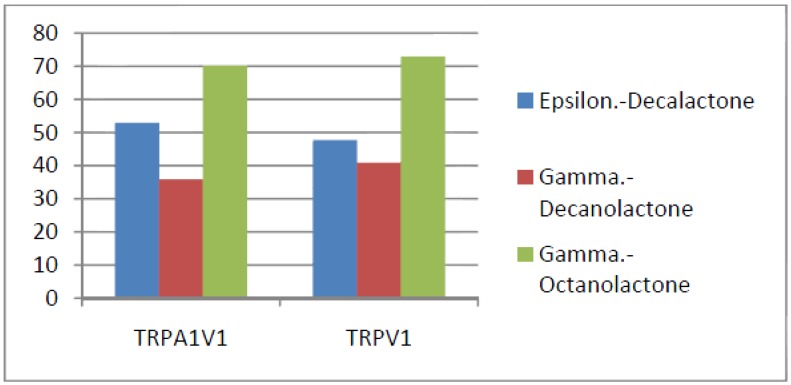
Examples of compounds that activate TRPV1 = TRPA1V1, but not TRPA1.

**Figure 7:**
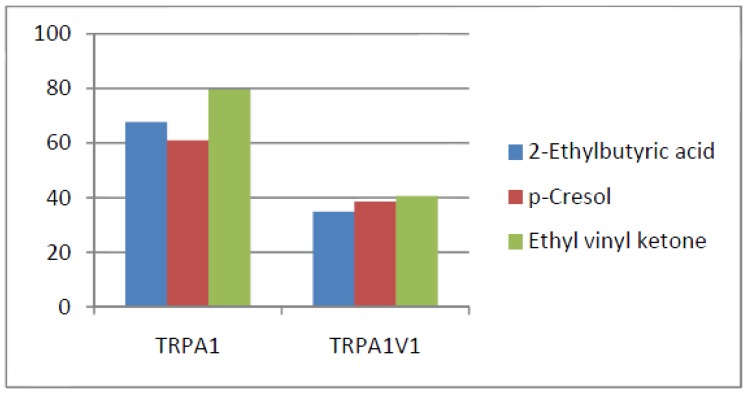
Examples of compounds that activate TRPA1 > TRPA1V1, but not TRPV1.

**Figure 8:**
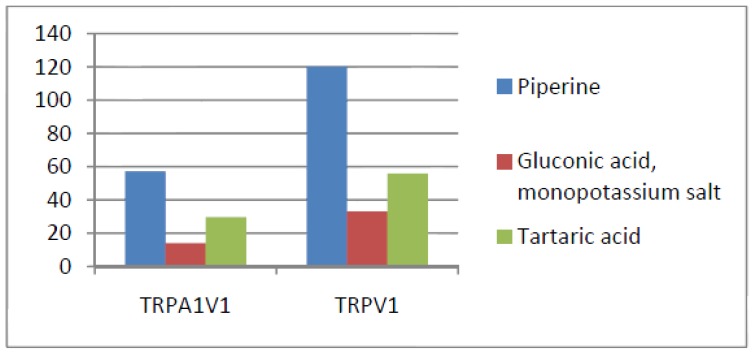
Examples of compounds that activate TRPV1 > TRPA1V1, but not TRPA1.

**Figure 9:**
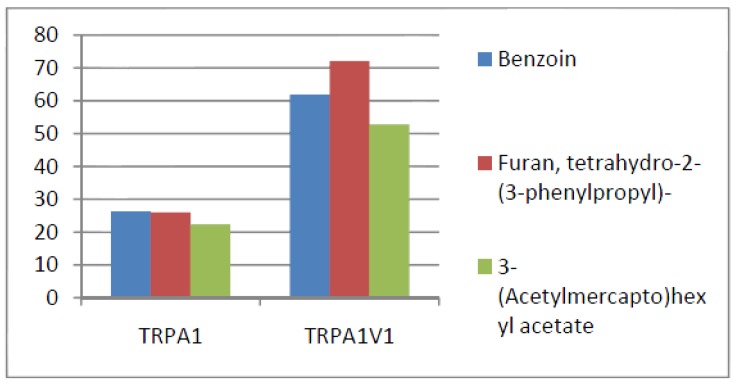
Examples of compounds that activate TRPA1 < TRPA1V1, but not TRPV1.

**Figure 10:**
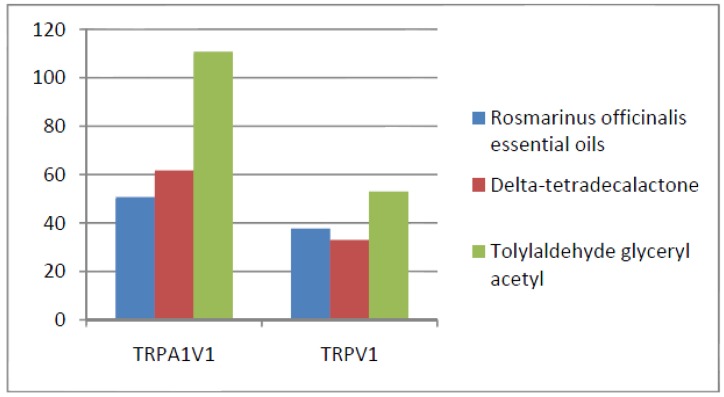
Examples of compounds that activate TRPV1 < TRPA1V1, but not TRPA1.

1. Compounds that activated all three channels with TRPA1 = TRPA1V1 > TRPV1: We identified 18 compounds that activated TRPA1 and TRPA1V1 equally but had less agonist activity at TRPV1 (Figure 3). This result indicates that the TRPA1 constituent of the TRPA1V1 was the key driver of binding of this class of compounds.
2. Compounds that activated all three channels with TRPA1 < TRPA1V1 = TRPV1: We identified 4 compounds that activated TRPV1 and TRPA1V1 equally but had less agonist activity at TRPA1 (Figure 4). This result indicates that the TRPV1 constituent of the TRA1V1 was the key driver of the binding of this class of compounds.
3. Compounds that activate TRPA1 = TRPA1V1 but not TRPV1: We identified 66 compounds that activated TRPA1 and TRPA1V1 to similar levels but did not activate TRPV1 (Figure 5). This result indicates that the TRPA1 component of the TRPA1V1 was the key driver of binding of this class of compounds.
4. Compounds that activate TRPV1 = TRPA1V1 but not TRPA1: We identified 6 compounds that activated TRPV1 and TRPA1V1 to similar levels but did not activate TRPA1 (Figure 6). This result indicates that the TRPV1 component of the TRPA1V1 was the key driver of binding of this class of compounds.
5. Compounds that activate TRPA1 > TRPA1V1 but not TRPV1: We identified 13 compounds that activated TRPA1 to a greater extent than TRPA1V1 but did not activate TRPV1 (Figure 7). This result indicates that the TRPV1 component of the TRPA1V1 had a negative effect on binding of the compound to TRPA1V1.
6. Compounds that activate TRPV1 > TRPA1V1 but not TRPA1: We identified 6 compounds that activated TRPV1 to a greater extent than TRPA1V1 but did not activate TRPA1 (Figure 8). This result indicates that the TRPA1 component of the TRPA1V1 had a negative effect on the binding of the compound to the TRPA1V1.
7. Compounds that activate TRPA1 < TRPA1V1 but not TRPV1: We identified 17 compounds that activated the TRPA1V1 to a greater extent than TRPA1 but with no activity at the TRPV1 receptor (Figure 9). This finding indicates that the TRPV1 association with TRPA1 had a positive effect on compound activation of TRPA1V1.
8. Compounds that activate TRPV1 < TRPA1V1 but not TRPA1: We identified 3 compounds that activate the TRPA1V1 to a greater extent than TRPV1 but with no activity at the TRPA1 receptor (Figure 10). This finding indicates that the TRPA1 association with TRPV1 had a positive effect on compound activation of the TRPA1V1.
9. Compounds that activate all three TRP receptors with complex effects on TRPA1, TRPA1V1 and TRPV1: We identified 2 compounds that activated TRPA1 > TRPA1V1 > TRPV1, 2 compounds that activated TRPA1 < TRPA1V1 < TRPV1, 2 compounds that activated TRPA1 = TRPV1 > TRPA1V1 and 7 compounds that activated TRPA1V1 > TRPA1 or TRPV1 < TRPA1V1 (Table 1). These findings demonstrated that compounds can behave uniquely when they interact with TRPA1 and TRPV1 compared to the TRPA1V1.
10. Compounds that activate either TRPA1 or TRPV1 or TRPA1V1 individually: We identified 1 compound that activated TRPA1 but not TRPA1V1 or TRPV1, 1 compound that activated TRPA1V1 but not TRPA1 or TRPV1 and 5 compounds that activated TRPV1 but not TRPA1V1 or TRPA1 (Table 2). This finding indicates that co-expression can change receptor activation and compound specificity.

**Table 1:** Examples of compounds that activated all three receptors with TRPA1V1 showing differential modulation.

Compound	TRPA1	TRPA1V1	TRPV1	Remarks
Ethyl 3-hydroxyoctanoate	97.29	76.90	28.05	Negative effect of V1
Geraniol	82.52	63.93	42.16	Negative effect of V1
5-Oxodecanoic acid	28.57	39.34	53.38	Negative effect of A1
Vanillylacetone	22.26	37.75	60.05	Negative effect of A1
2-Napphlaenthiol	66.3	85.2	120.3	Negative effect of A1
3-Methyl-5-propyl-2-cyclohexen-1-one	69.00	101.0	59.22	A1 and V1 additive
Benzaldehyde propylene glycol acetal	48.03	78.33	34.38	A1 and V1 additive
Methyl cyclohexanecarboxylate	45.50	85.17	69.51	A1 and V1 additive
4-Hydroxybenzaldehyde	80.4	163.2	123.1	A1 and V1 additive

**Table 2:** Examples of compounds that activate either TRPA1 or TRPA1V1 or TRPV1.

Compound	TRPA1	TRPA1V1	TRPV1
Propylparaben	93.2	2.1	1.6
Quercinitol	1.3	57.6	2.1
Malic acid	1.9	2.4	67.5
2-Methylbutyl isovalerate	1.7	2.1	75.3
2,4,5-Trimethyl-3-oxazoline	2.1	4.1	100.7
Heptaldehyde	4.3	2.4	65.3
2-Ethylfuran	1.2	1.8	58.7

#### 3.1.3. Activity of TRPA1, TRPAV1 and TRPV1 Agonists towards TRPV3 and TRPM8

All together 146 compounds including extracts, that activated one or more of TRPA1, TRPA1V1 and TRPV1 receptor were tested for their activity towards TRPV3 and TRPM8. As shown in Table 3, only one compound activated all five TRPV1, TRPA1V1, TRPA1, TRPM8 and TRPV3; one compound activated four receptors TRPV3, TRPA1, TRPA1V1 and TRPV1; one compound activated TRPA1, TRPA1V1 and TRPV3; one compound activated TRPA1V1, TRPV1 and TRPM8; three compounds activated TRPA1, TRPA1V1 and TRPM8. Thus, only seven out of 146 tested showed activation of TRPM8 and/or TRPV3.

**Table 3:** Activity of TRPA1, TRPAV1 and TRPV1 agonists towards TRPV3 and TRPM8.

Compound or Extract	TRPA1	TRPA1V1	TRPV1	TRPV3	TRPM8
L-Piperitone	45	68.4	0	0	81.6
(+/−)-Piperitone	90.1	109.1	53.3	0	45.6
DL-Menthol	54	43	0	0	78.4
Tannic acid	0	69.3	52.6	0	67.4
Eugenol	79	91.3	0	0	70.4
Beta-cyclohomocitral	81.4	66.8	61.7	76.5	70.7
Rosemarinus officinalis essential oils	37.7	50.7	0	85.6	0

## 4. Conclusions

We found 33 °C to be the preferred temperature for maintaining TRPV1 and TRPA1V1 cells in culture, probably resulting from temperature induced activation of TRPV1 which causes cellular toxicity. By screening over 600 materials we identified a number of compounds that activated TRPA1, TRPV1 and TRPA1V1, often with surprising results. In particular, we observed dramatic changes in TRP channel activation depending on whether the channels were expressed individually or were co-expressed, with the most surprising observation that the receptor profile can change an agonist to a non-agonist and *vice versa*. These results demonstrate that co-expression of TRPA1 and TRPV1 in the same cell can completely change the response of these channels to compounds, with the potential to completely change the sensation of the compounds in nerves. It will be interesting to probe structural features of compounds which result in observed unique differences in response towards TRPA1V1.

One of our interests in TRP channels is for the purpose of finding technologies to provide delightful sensorial attributes to consumer products, including taste modifications as well as to minimize adverse sensorial effects caused by essential functional components in our formulations. In this regard, TRPV1 and TRPA1 are key receptors in our sensory nerves from head to toe. Because these key sensorial receptors are often co-expressed in same sensory nerves, it is preferable to use cells that co-express these receptors for *in vitro* assessment of sensorial attributes of finished products as well as for discovery of novel sensates. Our results clearly demonstrate the value of using cells that co-express the two receptors, because in many instances the response seen with combined receptors is quite different from that predicted based on response to single receptors. Our screen has also identified compounds that uniquely activate TRPA1, TRPV1, or TRPA1V1. We plan to investigate their effect on sensory nerves. These compounds will be useful in isolating the trigeminal response in people, as impacted by activation of only one of these channels at a time. 

## References

[1] Bautista D.M., Jordt S.E., Nikai T., Tsuruda P.R., Read A.J., Poblete J., Yamoah E.N., Basbaum A.I., Julius D. (2006). TRPA1 mediates the inflammatory actions of environmental irritants and proalgesic agents. Cell.

[2] Birrell M.A., Belvisi M.G., Grace M., Sadofsky L., Faruqi S., Hele D.J., Maher S.A., Freund-Michel V., Morice A.H. (2009). TRPA1 agonists evoke coughing in guinea-pig and human volunteers. Am. J. Resp. Crit. Care Med..

[3] Caterina M.J., Schumacher M.A., Tominaga M., Rosen T.A., Levine J.D., Julius D. (1997). The capsaicin receptor: A heat-activated ion channel in the pain pathway. Nature.

[4] Bandell M., Story G.M., Hwang S.W., Viswanath V., Eid S.R., Petrus M.J., Earley T.J., Patapoutian A. (2004). Noxious cold ion channel TRPA1 is activated by pungent compounds and bradykinin. Neuron.

[5] Story G.M., Peier A.M., Reeve A.J., Eid S.R., Mosbacher J., Hricik T.R., Earley T.J., Hergarden A.C., Andersson D.A., Hwang S.W. (2003). ANKTM1, a TRP-like channel expressed in nociceptive neurons, is activated by cold temperatures. Cell.

[6] Jordt S.E., Bautista D.M., Chuang H.H., McKemy D.D., Zygmunt P.M., Högestätt E.D., Meng I.D., Julius D. (2004). Mustard oils and cannabinoids excite sensory nerve fibres through the TRP channel ANKTM1. Nature.

[7] McNamara C.R., Mandel-Brehm J., Bautista D.M., Siemens J., Deranian K.L., Zhao M., Hayward N.J., Chong J.A., Julius D., Moran M.M. (2007). TRPA1 mediates formalin-induced pain. Proc. Natl. Acad. Sci..

[8] Kedei N., Szabo T., Lile J.D., Treanor J.J., Olah Z., Iadarola M.J., Blumberg P.M. (2001). Analysis of the native quaternary structure of vanilloid receptor 1. J. Biol. Chem..

[9] Akopian A.N., Ruparel N.B., Jeske N.A., Hargreaves K.M. (2007). Transient receptor potential TRPA1 channel desensitization in sensory neurons is agonist dependent and regulated by TRPV1-directed internalization. J. Physiol..

[10] Akopian A.N., Ruparel N.B., Patwardhan A., Hargreaves K.M. (2008). Cannabinoids desensitize capsaicin and mustard oil responses in sensory neurons via TRPA1 activation. J. Neurosci..

[11] Macpherson L.J., Dubin A.E., Evans M.J., Marr F., Schultz P.G., Cravatt B.F., Patapoutian A. (2007). Noxious compounds activate TRPA1 ion channels through covalent modification of cysteines. Nature.

[12] Ruparel N.B., Patwardhan A.M., Akopian A.N., Hargreaves K.M. (2008). Homologous and heterologous desensitization of capsaicin and mustard oil responses utilize different cellular pathways in nociceptors. Pain.

[13] Staruschenko A., Jeske N.A., Akopian A.N. (2010). Contribution of TRPV1-TRPA1 interaction to the single-channel properties of the TRPA1 channel. J. Biol. Chem..

[14] Patil M.J., Jeske N.A., Akopian A.N. (2010). TRPV1 regulates activation and modulation of TRPA1 by Ca^2+^. Neurosience.

[15] Sadofsky L.R., Campi B., Trevisani M., Compton S.J., Morice A.H. (2008). Transient receptor potential vanilloid-1-mediated calcium responses are inhibited by the alkylamine antihistamines dexbrompheniramine and chlorpheniramine. Exp. Lung Res..

[16] Mitchell J.E., Campbell A.P., New N.E., Sadofsky L.R., Kastelik J.A., Mulrennan S.A., Compton S.J., Morice A.H. (2005). Expression and characterization of the intracellular vanilloid receptor (TRPV1) in bronchi from patients with chronic cough. Exp. Lung Res..

[17] Sadofsky L.R., Sreekrishna K., Morice A.H., Chung K.F., Widdicombe J. Characterisation of a HEK293 cell line permanently co-expressing the cough receptors Transient Receptor Potential Ankyrin 1 and Vanilloid 1 (TRPA1 and TRPV1). The Sixth London International Symposium on Cough: A translational approach.

[18] Smart D., Jerman J.C., Gunthorpe M.J., Brough S.J., Ranson J., Cairns W., Hayes P.D., Randall A.D., Davis J.B. (2001). Characterization using FLIPR of human vanilloid VR1 receptor pharmacology. Eur. J. Pharmacol..

[19] Zhang Y., Sreekrishna K., Lin Y., Huang L., Eickhoff D., Degenhardt D., Xu T. (2011). Modulation of Transient Receptor Potential (TRP) Channels by Chinese Herbal Extracts. Phytother. Res..

[20] Everaerts W., Gees M., Alpizar Y.A., Farre R., Leten C., Apetrei A., Dewachter I., van Leuven F., Vennekens R., de Ridder D. (2011). The capsaicin receptor TRPV1 is a crucial mediator of the noxious effects of mustard oil. Curr. Biol..

[21] Capasso R., Aviello G., Romano B., Borrelli F., de Petrocellis L., di Marzo V., Izzo A.A. (2012). Modulation of mouse gastrointesti­nal motility by allyl isothiocyanate, a constituent of cruciferous vegetables (Brassicaceae): Evidence for TRPA1-independent effects. Br. J. Pharmacol..

[22] Son H.J., Kim Y., Misaka T., Noh B.S., Rhyu M.R. (2012). Activation of the chemosensory ion channels TRPA1 and TRPV1 by hydroalcohol extract of *Kalopanax. pictus* leaves. Biomol. Ther..

