# Correction: Sadofsky, L.R., *et al.* Unique Responses are Observed in Transient Receptor Potential Ankyrin 1 and Vanilloid 1 (TRPA1 and TRPV1) Co-Expressing Cells. *Cells* 2014, *3*, 616-626

**DOI:** 10.3390/cells3040994

**Published:** 2014-10-27

**Authors:** Laura R. Sadofsky, Koti T. Sreekrishna, Yakang Lin, Renee Schinaman, Kate Gorka, Yogita Mantri, John Christian Haught, Thomas G. Huggins, Robert J. Isfort, Charles C. Bascom, Alyn H. Morice

**Affiliations:** 1 Cardiovascular and Respiratory Studies, The University of Hull, HU6 7RX, UK; E-Mails: Sadofsky@hull.ac.uk (L.R.S.); A.H.Morice@hull.ac.uk (A.H.M.); 2 The Procter & Gamble Company, Mason, OH 45040, USA; E-Mails: lin.y.2@Pg.com (Y.L.); schinaman.cr@pg.com (R.S.); gorka.k@pg.com (K.G.); mantri.y@pg.com (Y.M.); haught.c@pg.com (J.C.H.); huggins.tg@pg.com (T.G.H.); Isfort.rj@pg.com (R.J.I.); bascom.cc@pg.com (C.C.B.)

The authors wish to make the following corrections to this paper [1]:

In Table 2 on page 623, the Quercinitol activation value for TRPA1V1 should be 2.3 instead of 57.6. Quercinitol does not activate TRPA1V1. We thank Michael J.M. Fisher (University of Erlangen, Germany) for his feedback which helped us to review our result.

The authors would like to apologize for any inconvenience caused to the readers by these changes.
